# Address before the Lowndes County (Miss.) Medical Society, at the Anniversary Meeting, May 29, 1873

**Published:** 1873-08

**Authors:** J. W. M. Shattuck, W. L. Lipscomb


### ADDRESS BEFORE THE LOWNDES COUNTY (MISS.) MEDICAL SOCIETY, AT THE ANNIVERSARY MEETING, MAY 29, 1873.

BY DRS. J. W. M. SHATTUCK AND W. L. LIPSCOMB.

*Ladies and Gentlemen, Fellow Members of the Columbus and Lowndes County Medical Society:*

In accordance with the Constitution of our Society, it becomes my duty to use a portion of this hour in the delivery of the Annual Address, selecting subjects as I deem appropriate to the occasion. Permit me, then, to present a few thoughts upon *Medicine and Medical Ethics.*

From our standard authorities we gather the following brief epitome of the history of medical science:

The early history of medicine is dependent upon the unprinted legends of the past, handed down by tradition through many generations and centuries of years. It relied upon the superstition of the people for success, and its practice was confined to priests and magicians, who were regarded with great religious awe by those who applied to them for relief from diseases inflicted, as they believed, by the anger of the gods.

The most ancient physicians we read of were those who embalmed the body of Jacob by order of Joseph. Moses was a physician, skilled in all the arts of the Egyptians, and his description of some of the skin diseases of his day are lucid and accurate, and worthy the study of the modern practitioner. His prohibitions of certain kinds of unclean food are found to be in accordance with well-established laws of hygiene.

Later in the history of the Hebrew nation, the Levitical priesthood were the medical practitioners, and so continued until our Saviour's time, though many outside of the priesthood were followers of the healing art. We have a noted instance in the case of Luke, a learned Greek and proselyte to the Jewish faith, who earned that meed of distinction so highly prized by physicians of every age—the *sobriquet* of "the beloved physician."

The study of medicine was extensively pursued by the ancient Egyptians, but was so mingled with their priestly ceremonies, with the charms of the necromancers, with the incantations of witches, and with astronomical horoscopes, that their records were all lost or deemed unworthy of preservation.

To the Greeks belong the credit of first attempting to collect the crude materials of medical knowledge and reducing them to the proportions of a science, and of establishing medical schools for the propagation of their views, and the education of pupils devoted exclusively to the study and practice of medicine. Esculapius founded the three great schools of Cos, Snidos and Rhodes. He lived about seven or eight hundred years before Christ, and was reckoned the most eminent practitioner of his time. He continued to be revered after his death; in fact, he was ranked among the gods, and temples were built in his honor and for his worship. His works and the teachings of his schools were all lost, or have not come down to us with any credible authentication.

About four hundred and fifty years before Christ appeared one of the most remarkable men of his own, or any succeeding age—Hippocrates, the father of medicine—a man of extraordinary vigor and breadth of intellect, and possessed of a store of learning equaled only by the depositories of the literature and philosophy of his day. He added to the knowledge gathered from the association and instructions of Plato, and Xenophon, and Heroditus, a rich variety of information and experience, acquired by extensive travel and intercourse with all the philosophers, and physicians, and scientists of the surrounding countries. He traveled extensively in the practice of his profession at the call of the imperial families, and among the wealthy and distinguished aristocrats who needed medical assistance. He was an indefatigable student, an accurate observer, a clear and logical reasoner, and kept a faithful record of his experience and views. He wrote scores of volumes upon medical subjects, and carefully preserved all the accumulations of his research and wisdom. Thus, Hippocrates laid the foundations of medical science, and was emphatically the father of medicine. Surrounded, as he was, by schools of philosophy and science, and living in an age of intellectual friction, in which every attribute of mind was called into active and daily use, his theory of medicine partook of the nature of the age in which he lived.

He established what has since been called the *Dogmatic* School of Medicine, or a school which recognized *theory* as the basis of *practice.* He studied—first, health, or physiology; then, disease, or pathology; and lastly, remedies, or therapeutics. With what wisdom his plan of study was arranged, and how much in accord the demands of truth and success, let the recorded assent and indorsement of more than two thousand years of scrutiny and investigation attest.

The leading idea of this greatest of physicians was, a recognition of what he called *Nature,* both in health and disease, whose indications he carefully studied and endeavored to imitate in the practice of medicine. To him, also, is traced the principle of medication by contraries—"*contraria, contraribus, nudentur.*" medentur

Another great value of the Hippocratic writings consist in their accurate and numerous descriptions of the symptoms of disease, and their relation to prognosis. To his school we are indebted for the idea of crisis and critical days in disease, and for many valuable suggestions upon the laws and influence of hygiene.

The Hippocratic or Dogmatic School of Medicine was followed by an opposing school, established in Alexandria, under the lead of Serapion, which received the name of *Empiricism,* or the *Empirical School.* This school ignored *theory,* and regarded only experience. They adhered strictly to experience, to the accumulation of means of treatment simply by observation and experiment, independent of any physiological or pathological reasoning. They ignored the study of *why* or *how* a patient was cured, and confined themselves to *what* cured him. Their motto was, "*Facts, not ideas.*"

Rome afterwards became the seat of another school, established by Asclepiades, the friend of Cicero, called the *Medodist,* or Eclectic School. This school pursued an eclectic course, choosing and combining what they deemed best in other schools—thus endeavoring to avail themselves of the truths and means of practice from physiological reasoning or deduction, together with those acquired by simple experience or observation. They recognized all means for the cure of disease. They gave a reason, if they could, but were willing to accept an experiente which they could not explain. They consulted the philosophers, and they learned from the old women. Their motto was, "*Success.*" Broad and comprehensive in their views, liberal and charitable in their conduct, they soon took the world, and, for a long period, were the most popular practitioners of the healing art.

Thus were laid before the Christian era those three great plains, upon which the superstructure of our medical temple has been built. Thus were established the three great schools of medicine that, from that time to the present, have continued to divide the votaries of medical science—Dogmatism on one hand, Empiricism on the other, and Eclecticism making the intermediate link and connection of the whole structure. Each has contributed its share to the progress of the work, and offered fields of labor adapted to the peculiar character of the mind and constitution of the laborers engaged.

Hippocrates, with broad, reflective brow and eye of thought, stands proudly upon his pedestal, a worshiper of reason and abstract truth. He demanded that knowledge acquired by the observation of facts should be first reduced to theory, and then to practice. With a profound faith in the intellect of man, he claimed the capacity to learn all the conditions of life. The laws of being, the problems of physiology, the derangements of disease, the actions of medicine, were, in his judgment, primary and indispensable subjects of investigation—underlying which were the unchanging, eternal principles of medical truth. He saw in a physical fact nothing but the development or the application of a principle. He held the senses secondary to the mind, and was unwilling to occupy so narrow and contracted a sphere. He labored with assiduous zeal to establish medicine as science based upon theoretical truth.

Successors to so distinguished a leader, we notice those great original thinkers who, by reason and deduction, have established our systems of medicine, and have announced those wonderful discoveries that have given immortality to their names—Stahl, Hoffman, Bell, Bichat, Rosori, Broussias, Brown, Virchow, Brown-Sequard—philosophers and physiologists whose original researches and brilliant attainments have illuminated and adorned the temple of medicine.

The industrious experimenter at Alexandria, barely raising his head from its position of labor, and resting his knife upon the dead body he was dissecting, announces that, in the study of truth, we must consult experience rather than reason; that observation and experience cover all the knowledge ever acquired by man. Theories are fallacious; reason is defective; but truth can be demonstrated.

Here, on the field of Empiricism, some of the most arduous and intelligent toilers in medicine have labored, and here, too, have been made some of the most important and beneficent discoveries of the healing art. Here have congregated that vast host of investigators, with knife and tourniquet, with crucible and retort, with pestle and mortar, with microscope and stethoscope, with forceps and speculum, and every appliance of art—making surgery a science, developing chemistry, enlarging the Materia Medica, establishing anatomy, and recording the facts of therapy and clinical medicine. Here labored Laenec and Louis; Cooper, Velpeau and Mott; Chamberlain, Simpson and Sims; Harvey and Jenner, and hosts of the great discoverers of remedies and medical facts, whose names will be remembered while medical science endures.

The circulation of the blood, the discovery of chloroform and quinine, the boon of vaccination, and tens of thousands of remedies and alleviators of disease, owe their origin to this school of medicine.

The Eclectic School has been composed of men of calmer and more deliberative casts of mind, and with higher and juster powers of discrimination. In the main, these school men have lacked the fire of genius and the enthusiasm of research. Their province has been that of selection and combination. They are the great middle class of teachers, who receive the new truths gathered by solitary explorers, that dot, here and there, the far-off firmament of thought, and who bring up the rich gems dug in the mines of experiment, and, by their processes of elimination and deduction, of selection and combination, prepare them for practical and beneficent use.

But Nature, in her prodigal moods, has sometimes given to this class instances of that rare combination of being approaching almost to the God-like; in which brain and body seem but the complement of each other; with intellectual capacity able to soar into the illimitable fields of hypothesis; with imaginations that step with logical precision from the misty clouds of conjecture to the blazing worlds of truth; with senses as keen as the eye of an eagle and the ear of a stag; with brawny muscles and iron bones that never weary with labor or tire with toil. Giants they stand, with one foot upon the lofty dome of the temple at Cos, and the other buried amid the smoking furnaces and piling libraries that gather in the city of Alexandria; and, uplifting their proud forms in majestic grandeur, their heads reach close to the borders of the Eternal City, where, with intellects clear as the white light that radiates from the face of the Father and spirits—warm and sympathetic as the bleeding heart of the interceding Son—they hold converse with Deity, and hand down to the groping doubters and fainting millions of this sin-blind and disease-cursed world truths pure and fresh from the mind of God, and fruit and water from the trees and streams which are for the healing of the nations.

Looking across the chasm of the dark ages into the antique world, we find such a character personated in the noble Roman, Celsus, and in Galen, the scholarly and elegant Greek.

These men lived in the age when first the mind of man found that it had wings, and was every where, engaged in every description of flight, from the wildest fantasy of the imagination to the maturest developed systems of philosophy. They lived in an age when hypothesis and doubt mingled in strangest confusion with theorem and truth; when religion bowed before ten thousand gods, and superstition hung her shadowy pall around every altar, and a

*spiritism,* compounded of the *devilish* and the *human,* with a trace of the *divine,* pervaded the whole domain of the human intellect, and found expression in sorcery, and incantation, and amulet, and charm.

In such an age as this, when the theory of medicine partook of the mental habitudes of the philosopher, and the practice was on the lowest basis of the people's ignorance and superstition, these men studied and thought, and observed, selected and recorded all that was good, and profitable, and true; and for eight hundred years Galen was the presiding genius of medicine, and projected the influence of his views and opinions into every portion of the world. He was the standard of medical orthodoxy, and a dissent from his views consigned the doubter, at once, to a place among charlatans and quacks.

After the revival of letters, when Christianity and civilization had disjointed and scattered into fragments the wisdom of the ancients, and amid the readjustments which were going on every where in philosophy and science, in religion and art, and in medicine, as in every other department, two more great Eclectics arose, and brought order out of chaos, and placed medicine again in the road to substantial progress and development. The English Sydenham—not inaptly called the second Hippocrates—and Cullen, the great Scotch nosologist and Christian philosopher, squared our noble science with the philosophy of Bacon, with the religion of Christ, and with the advanced position of physical science.

Under these auspices medicine was established in America. Never did Eclecticism—or, to give it a name free from the odium of association with a late set of medical pretenders—never did Rational Empiricism find a more congenial soil than in the American mind. Providence, in its overruling direction, appears to have arranged and reserved our Western Continent for the development of those great conservative forces by which man was to be raised in the scale of being, and advanced, with steady and certain step, up to the highest conditions of happiness. We have here a country which, by the extent of her domain, the majesty of her mountains, and rivers, and lakes, and fruits, and the teeming products of her soil, suggests at once a scope for the mightiest capabilities. "No pent-up Utica contracts our powers." A land of liberty, with no

"Master" in its vocabulary, and in which authority is only a badge of office; a land of freedom! freedom from the shackles of opinion, ancient or modern—freedom from prescriptions in Latin or Greek—freedom from the edicts of royalty or the rules of priest-craft—freedom to think—freedom to work—every man according to his own individuality.

But it is "the utilitarian character of the American mind that distinguishes it from all others. Without much fondness for recondite subjects, or the injurious speculations of philosophers, our people have a keen perception of whatever ministers to the comforts and convenience of the species, and a power of adaption of which the world can afford no equal." They write "*cui bono*" upon every thing that comes within the scope of their vision or thought.

American medical teachers and practitioners share this characteristic in common with their countrymen. With a wisdom and precision that seems almost a part of Nature, they step up among the theories and principles of medicine, and choose those from which the surest and simplest deductions of practice may be drawn; they step down among the ten thousand articles of the Materia Medica, and bring up only such as are of known and positive virtue and efficacy. They understand Bacon, when he says: "If experience is not directed by theory, it is blind." They understand Trosseau, when he says: "Rationalism in medicine leads only to absurdities." But better, still, they understand the aphorism of Celsus, the great founder of their Eclectic system—an aphorism that ought to be written in letters of gold in every school and office of medicine: "*Medicine ought to be rational, but to draw its methods from the evident causes—all the obscure being removed, not from the attention of the artist, but from the practice of the art.*"

We are proud of American medicine, and would gladly enter the inviting field of portraiture, description and history; but the limits and design of our address prevent our entering so special a field. Our design has been to follow the elevation of medicine in its several *general* agencies, and its development into a stupendous whole; into the vast, magnificent science that is to-day the wonder of the world.

(TO BE CONTINUED.)

