# Errata

**Published:** 1873-09

**Authors:** 


*Errata.*

The reader will please make the following corrections in the address on Medicine and Medical Ethics, published in our August number, viz.: On page 469, for "nudentur," read "medentur"; page 470, for "Medodist," read "Methodist"; page 471, for "Rosoni," read "Rasori"; page 472, for "Father and spirits," read "Father of spirits"; page 474, for "injurious," read "ingenious."

